# Anthropometric and Biochemical Parameters in Relation to Dietary Habits as Early Indicator of Cardiovascular Impairment in Young Adult Cohort

**DOI:** 10.3390/ijerph17249208

**Published:** 2020-12-09

**Authors:** Nikolina Kolobarić, Maja Gradinjan Centner, Petar Šušnjara, Anita Matić, Ines Drenjančević

**Affiliations:** 1 Institute and Department of Physiology and Immunology, Faculty of Medicine Osijek, Josip Juraj Strossmayer University of Osijek, Josipa Huttlera 4, 31000 Osijek, Croatia; nbdujmusic@mefos.hr (N.K.); psusnjara1@gmail.com (P.Š.); acosic@mefos.hr (A.M.); 2 Scientific Centre of Excellence for Personalized Health Care, Josip Juraj Strossmayer University of Osijek, Trg Sv. Trojstva 3, 31000 Osijek, Croatia; 3 Department of Endocrinology, University Hospital Centre Osijek, Josipa Huttlera 4, 31000 Osijek, Croatia; majagradinjan@gmail.com

**Keywords:** diet, body mass index, cholesterol levels

## Abstract

Adjusted dietary assessment questionnaire was used to determine dietary habits of medical students which were related to biochemical and anthropometric markers of studied cohort. Thirty-seven young and healthy volunteers aged 19–28 years old entered the protocol and were divided according to sex and according to residence. Subjects were given questionnaires for tracking food/beverage consumption. Venous blood samples were taken after overnight fast (*n* = 32). Nutrient status and energy consumption were determined and analyzed. Study population had normal weight and body mass index (BMI). Biochemical characteristics were within normal reference range, while some participants had lipid profile disbalance. Men had significantly higher BMI than women. Average BMI was significantly higher in participants with elevated cholesterol levels compared to participants with normal cholesterol levels. Majority of participants consumed less than five meals per day with no major differences between students according to residence and sex. Men had significantly higher protein intake and consumed at least four meals daily compared to woman who had three or less meals daily with no differences in intake according to residence. Students with normal lipid profile consumed more carbohydrates than students with increased cholesterol. Results suggest that students with bad dietary habits have potentially higher risk for future cardiovascular problems, even before the onset of adverse effects.

## 1. Introduction

While optimal nutrition contributes to health, wellbeing, and normal development [1,2], an unbalanced, nutrient-poor diet accompanied by sedentary lifestyle and harmful habits, consequently, leads to greater risk of developing a variety of chronic diseases including obesity, diabetes, cardiovascular diseases, and cancer [3,4,5,6]. Higher body mass index (BMI) and weight problems in youth, usually linked to poor diet, are likely to cause hypertension and other cardiovascular problems together with reduced quality of life in future [7,8,9]. Further, according to Lupi et al. (2015) [10], there is also a visible change in student dietary habits present when living away from family. In our previous studies, medical students have been shown to generally eat less salt than general population, particularly women [11,12], which could suggest their better knowledge on needs to eat healthier.

Considering geographical location of Croatia, there are several types of local cuisines present alongside, Turkish (pastries, bread, and coffee), Hungarian (meat stews), Mediterranean (olive oil, fish, nuts), and Slavic (lard, dried meat) influences depending on the region [13]. The mediterranean diet (MD), characterized by consumption of whole grains, vegetables and fruits, olive oil, nuts, fish, and low amounts of local dairy products/red meat, has been claimed as a powerful tool in prevention of developing chronic diseases. Positive influences of MD include cardiovascular protection, metabolic and cognitive benefits, and protection against stroke, obesity, diabetes, cancer, and allergies [14,15,16,17,18]. On the other hand, consumption of deep-fried/roasted foods, alongside higher intake of saturated fatty acids and smoked products of animal origin, are typical for our research area [19]. Jelinić et al. (2009) [13] recorded highest prevalence of unhealthy dietary habits among general population (representative sample) in Eastern Croatia which is where our study group is located. Further, student population has certain specificities in their dietary habits such as irregular meals and higher energy intake, with women showcasing an overall better diet quality than men [20].

Accurate dietary assessment (DA) is essential for gathering data on food consumption, dietary habits and nutritional status at the population and individual level [21]. Alongside measurements of anthropometric and biochemical parameters and clinical examination, DA is a necessary tool in prediction of chronic disease’s risk and in overall health promotion [3,4,6,22]. Food Frequency Questionnaires (FFQ) and 24-h recalls (24HR) are the most frequently used methods in dietary and nutrition research [4,23] Therefore, the main objective of the present study was to assess dietary habits and health status in a sample of medical students’ population with an assembled dietary assessment questionnaire that is easy and fast to fill out by our volunteering participants. This questionnaire was adapted to presumed eating habits prevalent in study area [24,25]. Furthermore, we aimed to relate dietary habits to biochemical and anthropometric markers of studied cohort to evaluate potential risks for future development of cardiovascular or metabolic diseases.

## 2. Materials and Methods

### 2.1. Study Design and Participants

This was cross-section observational study. Participants visited our facility at two separate occasions. At the first appointment, subjects were given three identical forms for tracking food and beverage consumption for the past 24 h with the first page having blank fields left for date, name and surname, sex, height, and weight of the participant alongside instructions on how to fill the form. They were instructed to hand over filled-in forms to the researcher at the follow-up three weeks later. The first two forms refer to weekdays and the third one to weekend days. All subjects were informed by the researcher via text message/email about filling in the form for the day before, so they did not subconsciously alter their usual diet habits. Research was conducted during May 2019, at the Department of Physiology and Immunology, Faculty of Medicine in Osijek, Croatia.

Forty young healthy participants of both sexes (students of lower university study years) entered the study voluntarily. Three participants have withdrawn from the study in the first week while thirty-seven have continued to participate in the study. Exclusion criteria for participants were prior history of hypertension, coronary artery disease, diabetes mellitus, renal impairment, or cerebrovascular disease of peripheral blood vessels. Participants were divided according to sex and according to residence: native—students that are studying in hometown; non-native—students studying out of hometown This study was conducted according to the guidelines laid down in the Declaration of Helsinki and all procedures involving research study participants were approved by the Ethical Committee of the Faculty of Medicine, University of Osijek (CLASS: 602-04/20-08/07; Reg. No.:2158-61-07-20-25). Written informed consent was obtained from all subjects.

### 2.2. Dietary Assessment

Rather than using standardized forms, we adapted our questionnaire to presumed eating habits prevalent in study area with the possibility of choosing the offered answers in the form of tabular presentation rather than writing down everything by hand, in order to avoid boredom and reluctance of volunteers to participate. The form (in supplemental material of this manuscript; Table S1) is constructed as a table for the sake of simplicity and transparency. Table is divided into four main parts: Breakfast, Lunch, Dinner, and Snack. Each meal involves food and beverages. Meals are divided into: Fruits, Vegetables, Dairy Products, Meats, Carbs, Cereals, Beverages, Sweets, and Nuts. Method of preparation and portion sizes were taken into consideration depending on the type of food. The participant fills the table by placing a plus/tick symbol in the places provided in accordance consumption and preparation of food that day. After each meal there is a section called “Comments” where it is necessary to emphasize whether any dietary supplement was taken that could significantly affect or alter nutrient intake that day. If not present in the table, what was consumed can also be written in this section. Portion sizes are described as: small (1/2 of a cup/slice/piece); medium/normal portion (cup/slice/piece); large (2 cups/slices/pieces).

### 2.3. Laboratory Testing, Dietary Habits, and Anthropometric Measurements

Venous blood samples were taken after overnight fast at the first appointment and were analyzed for full blood cell count, plasma electrolytes (sodium, potassium, and calcium), iron, transferrin, creatinine, urea, fasting lipid profile including total cholesterol, high-density lipoprotein cholesterol (HDL cholesterol), low-density cholesterol (LDL-cholesterol), and triglycerides, fasting blood glucose and high-sensitivity C-reactive protein (hsCRP) using standard laboratory methods at the Department of Clinical Laboratory Diagnostics, University Hospital Osijek.

Nutrient status and energy consumption was determined and analyzed with Nutri Prog—software for nutritionists and dieticians at the Department of Endocrinology, University Hospital Osijek. BMI was calculated from information on body mass (kg) and height (m) which was obtained by researcher at first appointment. BMI was calculated and categorized based on WHO criteria for European population: Underweight <18.5 kg/m^2^; Normal weight 18.5–24.9 kg/m^2^; Pre-obesity 25.0–29.9 kg/m^2^; Obesity class I 30.0–34.9 kg/m^2^; Obesity class II 35.0–39.9 kg/m^2^; and Obesity class III > 40 kg/m^2^.

### 2.4. Statistical Analysis

Statistical analyses were performed using Microsoft Excel 2016 and Graph Pad Prism v6.01 software. T-test was used for differences among groups; *p* < 0.05 was considered significant. All results are expressed as average ± standard deviation (SD). Results on dietary habits were expressed as percentage (%) of adherence or failure to adhere to recommended guidelines. Chi–square test was performed for differences between groups regarding adherence to recommended guidelines; *p* < 0.05 was considered significant. Correlation coefficient was measured for relations between parameters.

## 3. Results

### 3.1. General and Biochemical Characteristics of Study Population

General characteristics of study population are shown in Table 1. All participants were young with no significant age difference according to sex. As expected, men were taller (*p* < 0.0001) and weighted (*p* < 0.0001) more than women. Participants were within normal BMI (kg/m^2^) with an average BMI of 23.6 ± 3.29 kg/m^2^. Men had significantly higher BMI than women (*p* = 0.007). One participant falls into the category of underweight (BMI = 18.3 kg/m^2^), while seven participants with a total average result 28.9 ± 3.24 kg/m^2^ belong to one of the obese categories. Five men and two women were moderately obese. Non-native students were significantly older than native (*p* = 0.0005). There were no significant average BMI, weight, and height differences between native and non-native students. Five native students were pre-obese (BMI = 29.7 ± 2.98 kg/m^2^), while one participant from non-native group was underweighted (BMI = 18.3 kg/m^2^).

Venous blood sampling was not successful in four women and one man; therefore, biochemical characteristics were analyzed for total of 32 participants. Biochemical characteristics of study population, as listed in Table 2, show that all measured parameters were within normal reference range. Men had significantly lower count of platelets compared to women (*p* = 0.003). As expected, women had significantly lower levels of urea (*p* = 0.009), creatinine (*p* < 0.0001), erythrocytes (*p* = 0.0002), hemoglobin (*p* < 0.0001), and hematocrit (*p* < 0.0001) compared to men, all within normal population reference range. There were no differences between sexes in serum electrolytes, iron, transferrin, fasting blood glucose, and hsCRP levels. Serum lipid profile parameters as well as red blood cell indices showed no differences between sexes. According to residence, all measured levels were in normal reference range without significant differences between native and non-native students.

### 3.2. Lipid Profile of Study Population

Lipid profile measurements of study population were in normal general population reference range, except slightly elevated LDL-cholesterol levels (3.06 ± 0.75 mmol/L). Women showed slightly higher average cholesterol levels than reference value (5.15 ± 1.04 mmol/L), while men remained in the recommended range (4.75 ± 0.89 mmol/L). LDL-cholesterol levels in women were above reference values with average of 3.20 ± 0.77 mmol/L. Native students had elevated average levels of cholesterol (5.21 ± 0.89 mmol/L) and LDL-cholesterol (3.25 ± 0.71 mmol/L) above reference values, while non- natives remained in the reference range.

### 3.3. Comparison of the Students Based on Lipid Profile

As shown in Table 3, reviewing the individual blood tests revealed that nearly half of the total number of students (*n* = 15), independently of residence or sex, have mildly elevated or high cholesterol levels (average of this subgroup was 5.78 ± 0.61 mmol/L) which was significantly higher compared to the rest of the participants (*n* = 17) that have individual values in the reference range (average value of cholesterol for this subgroup was 4.19 ± 0.49 mmol/L) (*p* < 0.0001).

Average cholesterol levels for this high-cholesterol critical group was 5.94 ± 0.72 mmol/L for women (*n* = 8) and 5.61 ± 0.42 mmol/L for men (*n* = 7). Triglycerides and LDL-cholesterol serum levels were significantly elevated in high- compared to low-cholesterol subgroup as well (*p* = 0.035 and *p* < 0.0001, respectively), while there was no difference in HDL-cholesterol levels. Moreover, average BMI was significantly higher in participants with elevated cholesterol levels (BMI = 25.3 ± 3.27 kg/m^2^) compared with participants that have normal cholesterol levels (BMI = 22.75 ± 2.84 kg/m^2^) (*p* = 0.013).

### 3.4. Dietary Habits of Study Population

Adherence of participants to the guidelines recommended by WHO in dietary habits of study cohort is shown in Table 4 (Food Based Dietary Guidelines, FBDG) [26,27].

Majority of study population (89%, *n* = 33) consumed less than five recommended meals per day with no major differences between sexes (Women 89%, *n* = 17 vs. Men 88%, *n* = 16) or residence (Native 95%, *n* = 21 vs. Non-native 80%, *n* = 12). Based on entire study population, 72% (*n* = 27) of participants failed to adhere to the guidelines for fruits and 76% (*n* = 28) for fish daily intake, while 64% (*n* = 24) of participants respected recommendations for protein, 86% (*n* = 32) for carbohydrate and 81% (*n* = 30) for vegetable intake. Daily intake of fluids, dairy products, whole grains, and nuts was equally distributed between participants who followed guidelines and those that failed to do so. Study population consumed more than recommended sweets and sodas, therefore they failed to adhere to the guidelines (Sweets: 75% of participants, *n* = 28; Sodas: 51% of participants, *n* = 19). More than a half of study population failed to drink enough fluids to meet the guidelines (51%, *n* = 19).

Large percentage of women consumed recommended amount of vegetables (94%, *n* = 18) and carbohydrates (78%, *n* = 15), while men mainly adhered to the guidelines on protein intake (77%, *n* = 14). Men also followed guidelines on carbohydrates (83%, *n* = 15) and vegetable (66%, *n* = 12) intake. Only 15% (*n* = 3) women and 33% (*n* = 6) men consumed satisfactory quantities of fish, while 21% (*n* = 4) women and 33% (*n* = 6) men included recommended amount of fruits in their diet. Overall, men had significantly higher protein intake (*p* = 0.02) compared to women.

According to residence, both groups mainly adhered to the guidelines for vegetable (native 90%, *n* = 20; non-native 66%, *n* = 10), carbohydrate (native 86%, *n* = 19; non-native 86%, *n* = 13) and protein (native 59%, *n* = 13; non-native 66%, *n* = 10) intake. Only 27% (*n* = 6) native and 26% (*n* = 4) non-native students included recommended amount of fruits in their diet. Fish was also among foodstuffs that was under-consumed in both groups, with only 22% (*n* = 5) of native and 26% (*n* = 4) of non-native students following the guidelines. Native students (86%; *n* = 19) consumed sweets more than recommended daily intake.

Three or less meals per day was consumed by 56% (*n* = 21) of study population, with major part of that being women (73%, *n* = 14, *p* = 0.033) while men consumed at least four meals per day (50%, *n* = 9, *p* = 0.027). Native students mainly consumed three or less meals per day (63%, *n* = 14), while non-natives students consumed five or more meals (20%, *n* = 3) or at least four meals (33%, *n* = 5) with prevailing three or less meals per day (46%, *n* = 7). More than half of non-native students consumed at least four meals per day which is not the case in native group.

In subgroup with elevated cholesterol levels, 61% of participants (*n* = 8) consumed at least four meals per day which is more meals throughout the day compared to the rest of the participants. On the other hand, 52% (*n* = 9) of participants with cholesterol levels in reference range consumed three or less meals per day. Moreover, participants with elevated cholesterol levels mostly followed guidelines for dairy products (66%, *n* = 10) and proteins (66% *n* = 10), while participants with normal cholesterol levels shown significantly higher intake of carbohydrates (94%, *n* = 16, *p* = 0.019).

### 3.5. Correlation Analysis

Correlation analysis was performed between biochemical parameters, anthropometric parameters, and dietary habits of study population. Based on study population, a significant positive correlation between hematocrit values and frequency of vegetable consumption (*r* = 0.594, *p* = 0.0004) was found. Significant positive correlation was also found between number of meals consumed and frequency of fruit consumption (*r* = 0.534, *p* = 0.0008).

According to residence, in non-native group a significant negative correlation was found between triglyceride levels and frequency of fruit (*r* = −0.556, *p* = 0.04) or vegetable (*r* = −0.685, *p* = 0.009) consumption, as well as between age of participants and frequency of sweets consumption (*r* = −0.569, *p* = 0.03). In native group, age of participants correlated positively to cholesterol levels (*r* = 0.544, *p* = 0.01). Women’s BMI correlated positively to cholesterol (*r* = 0.819, *p* = 0.0001) and LDL-cholesterol (*r* = 0.856, *p* < 0.0001) levels, as well as age of female participants and their body weight (*r* = 0.643, *p* = 0.003) did. Men’s triglyceride levels correlated negatively to frequency of vegetable consumption (*r* = −0.753, *p* = 0.0005) which was also found in high-cholesterol subgroup (*r* = −0.713, *p* = 0.003). Additionally, in high-cholesterol group, positive correlation was found between number of meals and frequency of carbohydrates (*r* = 0.616, *p* = 0.01), dairy (*r* = 0.546, *p* = 0.03), sweets (*r* = 0.673, *p* = 0.006), and coffee (*r* = 0.552, *p* = 0.03) consumption.

## 4. Discussion

A dietary assessment questionnaire in present study was designed to examine students’ dietary habits but also to provide the insights into differences between sexes and differences in habits of students in regard of residence status in the city of studying. In addition, we analyzed data on the dietary habits of students with inadequate serum lipid profile levels. Dietary habits in Croatian students were addressed earlier [24,25], but those were mainly large observational studies focusing on nutritional status rather than its relation to anthropometric and biochemical parameters of a small, isolated population. Thus, in present study we have performed additional analyses on these parameters that reflect cardiovascular status in relation to dietary habits.

Main findings of the present study were that students skipped meals and had quite irregular intake of carbohydrates, sweets, and sodas. Furthermore, a major part of men followed recommendations regarding protein intake which may characterize men as “protein type”. On the other hand, contrary to popular opinion that women are “carb type” and usually find resort in refined sugars, almost all women in our study adhered to guidelines on carbohydrate and vegetable intake. Native students who live in family home consumed vegetables on a regular basis, while non-natives relied on whole grains and proteins.

Interestingly, students with normal cholesterol blood levels consumed a high-carbohydrate diet, while high cholesterol subgroup consumed more proteins and dairy products. Results of present study suggest that students consumed extremely low amounts of omega-3 rich foods (fish and nuts), which are rather discouraging, considering the known beneficial effect of polyunsaturated fatty acids on microvascular reactivity, oxidative stress reduction, and lipid profile repairment [28,29,30,31]. Consequently, there are only few similarities between diets of participants in present study and elements of Mediterranean diet.

According to WHO, basics of good dietary habits are at least five portions of fruit and vegetables per day (excluding starchy roots), total fat intake reduced to less than 30% (preferably saturated and trans-fats replaced with unsaturated), reduced salt intake to less than 5 g per day and intake of free sugars reduced to <5% through five or more meals per day (three main meals and two snacks) [26,32]. In present study, students’ average intake of three meals per day is below recommended intake. Only a small percentage of medical students consumed five or more meals daily and those were prevalently non-native students.

Young adults, specifically university population has been shown to being prone to poor dietary choices [33,34]. Quality of lifestyle/food consumed is varying greatly between those studying away from home and those studying in home residence; nevertheless, often both groups tend to reach for junk food in stressful environment [10]. Therefore, native students in our study expectedly consumed more vegetables in the comfort of a family home, while non-native students had more discipline in taking regular daily meals and consumed fewer sweets as they got older.

Unhealthy dietary choices and nutrition, physical inactivity, cigarette smoking, and heavy alcohol consumption are harmful habits contributing to increasing prevalence of obesity, cardiovascular disease, type 2 diabetes, hypertension, and several types of cancer in all industrialized countries [6,35,36,37,38]. The most common unhealthy dietary habits among students are irregular/skipped meals paired with fried food consumption, low fruits, and vegetables intake and frequent snacks in between meals [39], which is in accordance with our results. Male students are usually shown more prone to being overweight and obese when compared to female students [40,41]. Results of our present study point in the same direction since there was a significant negative correlation found between triglyceride levels and vegetable/fruit consumption of our study participants. Although, in our research elevated cholesterol/LDL-cholesterol levels and weight problems were correlated positively to age of participants, problems like obesity accompanied by disorders such as insulin resistance and hyperlipidemia usually affect individuals since childhood; therefore, it is never too early to take preventive measures [42].

Hyperlipidemia is one of the main causes of increased risk for cardiovascular diseases. Hypercholesterolemia and hypertriglyceridemia (can occur individually or in combination) are the most common disorders globally, and can be prevented by certain lifestyle changes such as regular exercise, low-fat diet, and consumption of food rich in fiber [6,43,44,45]. As suggested by Tsai et al. (2020) [46], there is a decreased incidence of CVD later in life when leading a healthy lifestyle in young adulthood. A shift to a healthier lifestyle is certainly beneficial for all age groups, but in this particular age group it serves as a prevention measure above all. Average biochemical parameters and lipid profile analysis of our study population were in reference range; however, when evaluating individual results, it was indicated that almost half of participants had elevated cholesterol and LDL-cholesterol levels above the reference values. According to Pletcher et al. (2010) [47], even the modest rises in LDL-cholesterol are associated with significantly higher risk of atherosclerosis. LDL-cholesterol levels during young adulthood are highly correlated with lipid levels later in life. As a result of non-optimal lipid levels, atherosclerotic changes begin to occur which is associated with coronary artery diseases later in life. A study by Zhang et al. (2019) [48] with a total sample size of more than 36,000 individuals confirms that higher cholesterol levels (especially LDL-cholesterol) and exposure to high blood pressure during young adulthood are highly associated with increased CVD risks later in life.

Subjects in the study that had elevated levels of cholesterol had also high intake of foods of animal origin such as proteins and dairy products. With increase in number of meals, this group also simultaneously consumes more carbohydrates, sweets, and coffee. This is in accordance with studies reporting that excessive consumption of meat and high-fat dairy products alter lipid levels in a negative context [49,50,51]. Further, as stated in results section, this group also has significantly higher BMI values compared to the rest of participants. Similarly, positive correlation between serum cholesterol levels and BMI was recently reported by Nwaiwu and Ibe (2015) [52] in children and Laclaustra et al. (2018) [53] in adults. Furthermore, in present study seven participants were overweight. Approximately 23% of women and 13% of men gain more than 20 kg between ages of 18 and 55 years [54]. Unhealthy diet and physical inactivity were more common among those who gained more weight through years, and so was the incidence of type 2 diabetes, hypertension, and other CDV diseases. Hypertension is five times more frequent among obese and overweight people than normal weighted people; therefore, overweight/obesity is contributing to a global increase in cardiovascular disease [55]. Weight gain occurs more frequently during early to middle adulthood with greater weight gain among women being explained by weight retention after childbirth [56]. This can explain why in our study men were leading in being overweight, considering the fact that involved women have not gone through pregnancy and childbirth just yet.

To grasps specificity of local cuisine that may influence results of dietary assessment, we have assembled a dietary assessment questionnaire in form of a table with offered answers regarding food items, portions, and preparation methods in accordance with these specificities and assumed dietary habits of students in the research area. It is based on what food items do students consume and do they follow recommended guidelines rather than exact amount in grams, milliliters etc. Instructions on filling out the forms were in accordance with standard three-day food records which note everything consumed during time period of three days; two questionnaires for weekdays and one for weekend day (avoid celebrations and similar occasions [4,5,57]. Its adjustment compared to FFQ and 24 h is a simplification of the standardized form with offered options for foods, portions and preparation, in order to avoid hand writing and reduce longevity of filling the forms. Questionnaire used in present study is different from standard FFQ, which is usually displayed as a list of items [3,58], in a way it takes into account the method of preparation and thus provides more information. Our questionnaire is closest to standard 24 h method in terms of gathered information on the type of food and its characteristics, preparation method and quantity consumed [21], although we mainly emphasized accordance of participants with general guidelines on intake for our area of research.

Lately, efforts are being made in the direction of creating novel approaches for improvement of the quality of life like metabolic diet typing and functional foods. Functional foods have specific health benefits through improvement/restoration of nutritional status and can be used as a step towards prevention of chronic disease development in the future [1]. For example, n-3 PUFAs supplements and in functional food have been associated with increased anti-inflammatory and antioxidant function and may provide other benefits in diabetes and obesity prevention [59,60,61]. Currently, the progress is being made in this field through studying the effect of human consumption of n-3 PUFAs enriched hen eggs on microvascular reactivity, lipid profile, oxidative stress, and antioxidative capacity [29,30,62]. Results of this study suggest that students consume extremely low amounts of n-3 PUFAs rich foods (fish and nuts) which are rather discouraging considering the mentioned beneficial effect on overall health. Tomić et al. (2014) [63] reported that there is a certain interest but also confusion regarding functional foods among younger consumers (18–30 years old) mostly related to lack of information and guidance. They found that young adults mostly evaluate functional foods on the basis of quality/price ratio and taste, which is not as discouraging as there is an interest present. It is a rather good starting point in an attempt to educate younger generation on wellbeing and overall health optimization.

Limitation of the study was that we have not evaluated physical activity of our participants, assuming that it is similar, due to the fact that all students are from general medical students’ population.

## 5. Conclusions

Present study demonstrated similarities and differences in dietary habits in sampled student population, depending on sex and residence. One of the outcomes is the assessment questionnaire that was assembled for the purpose of the study. Furthermore, results suggest that poor dietary habits change health status in a negative context in young and seemingly healthy individuals even before the noticeable manifestation of detrimental effect of it. These impairments potentially increase the risk for future cardiovascular diseases, especially in those individuals with inadequate serum lipid profile.

## Figures and Tables

**Table 1:** General characteristics of study population.

Parameter	All Participants	Women	Men	*p*	Native	Non-Native	*p*
*n*	37	19	18	-	22	15	-
Age (years)	23.9 ± 2.4	24.4 ± 2.3	23.4 ± 2.5	0.315	25 ± 2.3	22.2 ± 1.3	0.0005 *
Weight (kg)	72.7 ± 13.2	63.21 ± 8.85	82.78 ± 8.74	<0.0001 *	73.27 ± 12.45	71.93 ± 14.58	0.777
Height (m)	1.75 ± 0.09	1.68 ± 0.06	1.83 ± 0.06	<0.0001 *	1.74 ± 0.09	1.77 ± 0.09	0.485
BMI (kg/m^2^)	23.6 ± 3.29	22.5 ± 3.24	24.7 ± 3.02	0.007 *	24.1 ± 3.63	22.7 ± 2.62	0.395
Underweight	18.3	18.3	-	-	-	18.3	-
Pre-obesity	28.9 ± 3.24 (*n* = 7)	29.8 ± 3.63 (*n* = 2)	29.3 ± 2.75 (*n* = 5)	0.571	29.7 ± 2.98 (*n* = 5)	27 ± 2.62 (*n* = 2)	0.285

Results are expressed as mean ± standard deviation (SD). *n*—number of participants; BMI—Body Mass Index. Student *t*-test, significance level *p* < 0.05 * Women vs. Men; Native vs. Non-native.

**Table 2:** Biochemical characteristics of study population.

Parameter	All Participants	Women	Men	*p* *	Native	Non-Native	*p* *	Reference Range
*n*	32	15	17	-	19	11	-	-
Urea (mmol/L)	5.5 ± 1.31	4.8 ± 0.94	6.08 ± 1.34	0.009 *	5.13 ± 1.13	6.11 ± 1.46	0.067	2.8–8.3
Creatinine (µmol/L)	81.41 ± 17.34	67.13 ± 8.83	94 ± 14.43	<0.0001 *	78.16 ± 15.63	89.27 ± 18.12	0.121	49–90
Sodium (mmol/L)	138.03 ± 2.06	137.33 ± 2.02	138.65 ± 1.93	0.139	138.05 ± 1.96	138 ± 2.45	0.457	137–146
Potassium (mmol/L)	4.18 ± 0.24	4.23 ± 0.24	4.14 ± 0.24	0.295	4.18 ± 0.27	4.16 ± 0.22	0.948	3.9–5.1
Calcium (mmol/L)	2.43 ± 0.07	2.41 ± 0.06	2.45 ± 0.07	0.071	2.45 ± 0.06	2.42 ± 0.07	0.262	2.14–2.53
Iron (umol/L)	18.22 ± 5.97	18.51 ± 5.85	17.96 ± 6.25	0.395	18.18 ± 6.74	17.3 ± 4.69	0.88	8.0–30.0
Transferrin (g/L)	2.84 ± 0.45	2.94 ± 0.51	2.74 ± 0.38	0.299	2.91 ± 0.47	2.68 ± 0.42	0.168	2.00–3.60
Fasting blood glucose (mmol/L)	4.76 ± 0.68	4.66 ± 0.42	4.84 ± 0.84	0.758	4.88 ± 0.78	4.56 ± 0.47	0.299	4.2–6.0
hsCRP (mg/L)	1.73 ± 2.06	2.09 ± 2.75	1.41 ± 1.18	0.933	1.79 ± 2.16	1.85 ± 2.09	0.846	<5.00
Cholesterol (mmol/L)	4.94 ± 0.97	5.15 ± 1.04	4.75 ± 0.89	0.439	5.21 ± 0.89	4.65 ± 1.01	0.127	<5.00
Triglycerides (mmol/L)	1.15 ± 0.75	1.03 ± 0.33	1.25 ± 0.98	0.948	1.08 ± 0.27	1.32 ± 1.24	0.321	<1.70
HDL cholesterol (mmol/L)	1.52 ± 0.36	1.59 ± 0.31	1.46 ± 0.39	0.265	1.59 ± 0.36	1.45 ± 0.37	0.491	>1.20
LDL cholesterol (mmol/L)	3.06 ± 0.75	3.2 ± 0.77	2.93 ± 0.73	0.275	3.25 ± 0.71	2.84 ± 0.79	0.143	<3.00
Leukocytes (x10E9/L)	6.15 ± 1.38	6.52 ± 1.35	5.82 ± 1.36	0.167	6.36 ± 1.29	6.09 ± 1.49	0.504	4.4–11.6
Platelets (x10E9/L)	245.81 ± 57.68	271.25 ± 66.76	219.06 ± 31.66	0.003 *	255.84 ± 65.35	232.09 ± 46.35	0.197	178–420
Erythrocytes (x10E12/L)	4.75 ± 0.34	4.53 ± 0.23	4.94 ± 0.31	0.0002 *	4.74 ± 0.34	4.82 ± 0.35	0.532	4.07–5.42
Hemoglobin (g/L)	141.16 ± 11.27	133.13 ± 8.07	148.24 ± 8.7	<0.0001 *	141.63 ± 11.12	142.91 ± 11.22	0.747	118–149
Hematocrit	0.41 ± 0.03	0.39 ± 0.02	0.43 ± 0.02	<0.0001 *	0.41 ± 0.03	0.41 ± 0.03	0.796	0.354–0.450
MCV (fL)	87.08 ± 3.69	86.56 ± 3.46	87.54 ± 3.94	0.449	87.45 ± 3.66	86.85 ± 4.09	0.714	76.5–92.1
MCH (pg)	29.74 ± 1.4	29.42 ± 1.36	30.02 ± 1.42	0.219	29.89 ± 1.45	29.63 ± 1.45	0.59	24.3–31.5
MCHC (g/L)	341.5 ± 5.79	339.93 ± 5.16	342.88 ± 6.12	0.114	341.68 ± 6.11	341.27 ± 5.92	0.948	304–346
RDW-CV (%)	13.9 ± 0.79	13.63 ± 0.96	14.15 ± 0.52	0.281	13.74 ± 0.86	14.19 ± 0.69	0.342	9.0–15.0
MPV (fL)	10.54 ± 0.54	10.33 ± 0.47	10.72 ± 0.54	0.059	10.49 ± 0.62	10.61 ± 0.45	0.635	7.0–10.4

Results are expressed as average ± standard deviation (SD). *n*—Number of participants; hsCRP—high sensitive C-reactive protein; HDL—high density lipoprotein; LDL—low density lipoprotein. Student *t*-test; significance level *p* < 0.05 * Women vs. Men; Native vs. Non-native. Reference range—general population.

**Table 3:** Comparison of lipid profile in high- and low-cholesterol subgroups.

Parameter	High-Cholesterol Subgroup	Low-Cholesterol Subgroup	Reference Range	*p*
*n*	15	17	-	-
BMI (kg/m^2^)	25.3 ± 3.27	22.7 ± 2.84	18.5–24.9	0.013 *
Cholesterol (mmol/L)	5.78 ± 0.61	4.19 ± 0.49	<5.00	<0.0001 *
Triglycerides (mmol/L)	1.33 ± 0.95	0.99 ± 0.49	<1.70	0.035 *
HDL cholesterol (mmol/L)	1.57 ± 0.37	1.48 ±0.35	>1.20	0.569
LDL cholesterol (mmol/L)	3.72 ± 0.49	2.47 ±0.33	<3.00	<0.0001 *

Results are expressed as average ± standard deviation (SD). *n*—number of participants; BMI—Body Mass Index; HDL—high density lipoprotein; LDL—low density lipoprotein. Student *t*-test. significance level *p* < 0.05 *. High-cholesterol vs. Low-cholesterol. Reference range—general population.

**Table 4:** General dietary habits of study population.

Parameter	Study Population	Women	Men	*p*	Native	Non-Native	*p*	HC	LC	*p*
Average meals per day ± SD	3.49 ± 0.77	3.32 ± 0.75	3.66 ± 0.77	-	3.36 ± 0.66	3.67 ± 0.89	-	3.47 ± 0.83	3.65 ± 0.79	-
Five or more meals per day	10.81	10.53	11.11	0.954	4.55	20	0.137	7.69	17.65	0.349
At least four meals per day	32.43	15.79	50	0.027 *	31.82	33.33	0.923	53.84	29.41	0.314
Three or less meals per day	56.76	73.68	38.89	0.033 *	63.64	46.67	0.306	38.46	52.94	0.723
Adherence to the recommended guidelines for daily intake of fruit units	27.03	21.05	33.33	0.400	27.27	26.67	0.967	20	29.41	0.539
Adherence to the recommended guidelines for daily intake of protein units	64.86	42.11	77.78	0.027 *	59.09	66.67	0.641	66.67	58.82	0.647
Adherence to the recommended guidelines for daily intake of carbohydrate units	86.49	78.95	83.33	0.733	86.36	86.67	0.979	60	94.12	0.019 *
Adherence to the recommended daily fluid intake guidelines	48.65	42.11	55.56	0.413	50	46.67	0.842	40	52.94	0.464
Adherence to the recommended guidelines for daily intake of dairy products	51.35	52.63	52.94	0.872	45.45	60	0.385	66.67	41.18	0.469
Adherence to the recommended guidelines for daily intake of vegetable units	81.08	94.74	66.67	0.063	90.91	66.67	0.065	80	82.35	0.865
Adherence to the recommended guidelines for daily intake of whole grains	43.24	42.11	44.44	0.886	31.82	60	0.089	46.67	35.29	0.513
Adherence to the recommended guidelines for daily intake of fish	24	15.8	33.3	0.214	22.73	26.67	0.784	13.33	29.41	0.272
Adherence to the recommended guidelines for daily intake of nuts	45.95	36.84	55.56	0.254	18.18	26.67	0.538	53.33	47.06	0.723
Failure to adhere to the recommended guidelines for daily intake of sweets	75.68	73.68	77.78	0.772	86.36	60	0.066	66.67	70.59	0.811
Failure to adhere to the recommended guidelines for daily intake of sodas	51.35	42.11	61.11	0.248	59.09	40	0.254	46.67	64.71	0.305

Results are expressed as percentage (%) of participants regarding consumption of food/beverages in terms of recommended guidelines. HC—high cholesterol; LC—low cholesterol. Chi-square test. Significance level *p* < 0.05 *. Women vs. Men; Native vs. Non-native; High-cholesterol vs. Low-cholesterol.

## Supplementary Materials

The following are available online at https://www.mdpi.com/1660-4601/17/24/9208/s1, Table S1: Dietary Assessment Form.
