# Supplementary material for: Anthropometric and Biochemical Parameters in Relation to Dietary Habits as Early Indicator of Cardiovascular Impairment in Young Adult Cohort
Source: Int J Environ Res Public Health. 2020 Dec 9;17(24):9208. doi: 10.3390/ijerph17249208 (PMC7764557; doi:10.3390/ijerph17249208)
Supplement: Supplementary file 1 [file ijerph-17-09208-s001.pdf]

**DATE: ___________**

**24 Hour Food Recall**

- FORM-

**FIRST NAME/ LAST NAME_____________________________**

**DATE OF BIRTH: ____________**

**SEX:** F M

**HEIGHT: _______________**

**WEIGHT: ______________**

At the first appointment subjects were each given three identical forms for tracking food and beverage consumption for the past 24 hours. The first two forms refer to weekdays and the third one to weekend days. All subjects were informed by the researcher via text message/e-mail about filling in the form for the day before so they don't subconsciously alter their usual diet habits. The form itself is constructed as a table for the sake of simplicity and transparency. Table is divided into four main parts: **BREAKFAST**, **LUNCH**, **DINNER** and **SNACK**. Each meal involves food and beverages. Meals are divided into: **FRUIT** (fresh / dry); **VEGETABLES** (fresh / frozen / cooked / baked); **DAIRY PODUCTS** (milk / cheese / yogurt); **MEAT** (white meat /red meat /fish / processed meat products); **CARBS** (bread / pasta / bagels / potatoes/ rice); **CEREALS** (oats /corn cereal / sweetened cereal); **BEVERAGES** (water / mineral water / coffee / coffee with milk/ tea / natural juice / soda / alcohol); **SWEETS** (chocolate / chips / cookies / cake); **NUTS** (almonds / hazelnuts / walnuts). Method of preparation and portion sizes were taken into consideration depending on the type of food. The subject fills the table in such way as he/she places a plus/tick symbol in the places provided for it in accordance with what he/she consumed that day and how it was prepared. After each meal there is a section called **COMMENT** where it is necessary to emphasize whether the subject was taking any dietary supplement or anything that could significantly affect or alter nutrient intake that day. If not present in the table, what was consumed can also be written in this section. If any of the meals are skipped also indicate.

*Portion sizes are described as: small (1/2 of a cup / slice / piece); medium/normal (cup / slice / piece); large (2 cups / slices / pieces).

**Way of consumption (dairy products / nuts): separately – a cup of milk, a piece of cheese, a handful of almonds; as part – cereals with milk, sandwich with ham and cheese, cereals with nuts.

| | | | | | | | | | |
|---|---|---|---|---|---|---|---|---|---|
| BREAKFAST | DAIRY PRODUCTS | | WAY OF CONSUMPTION | | PORTION SIZE | | | | |
| | | DIVISION | Separately | As part | Small | Medium | Large | | |
| | | Cheese | | | | | | | |
| | | Milk | | | | | | | |
| | | Yoghurt | | | | | | | |
| | | ___________ | | | | | | | |
| | MEAT | | PREPARATION | | | | PORTION SIZE | | |
| | | DIVISION | Raw | Cooked | Baked | Fried | Small | Medium | Large |
| | | White meat | | | | | | | |
| | | Red meat | | | | | | | |
| | | Fish | | | | | | | |
| | | Meat products | | | | | | | |
| | | _________ | | | | | | | |
| | CARBOHYDRATES | | PREPARATION | | | | PORTION SIZE | | |
| | | DIVISION | Raw | Cooked | Baked | Fried | Small | Medium | Large |
| | | Bread | | | | | | | |
| | | Bagels | | | | | | | |
| | | Pasta | | | | | | | |
| | | Potatoes | | | | | | | |
| | | Rice | | | | | | | |
| | FRUIT | | PREPARATION | | PORTION SIZE | | | | |
| | | DIVISION | Fresh | Dried | Small | Medium | Large | | |
| | | Apple/Pear | | | | | | | |
| | | Banana | | | | | | | |
| | | Berries | | | | | | | |
| | | ___________ | | | | | | | |
| | VEGETABLES | | PREPARATION | | | | PORTION SIZE | | |
| | | DIVISION | Fresh | Frozen | Cooked | baked | Small | Medium | Large |
| | | Legumes | | | | | | | |
| | | Tuberous/Roots | | | | | | | |
| | | Cabbage | | | | | | | |
| | | Bulbs | | | | | | | |
| | | Pumpkins | | | | | | | |
| | | _________ | | | | | | | |
| | CEREALS | | PORTION SIZE | | | | | | |
| | | DIVISION | Small | Medium | Large | | | | |
| | | Oats | | | | | | | |
| | | Corn | | | | | | | |

| | | | | | | | | |
|---|---|---|---|---|---|---|---|---|
| | | Sweetened | | | | | | |
| | | ________________ | | | | | | |
| | NUTS | | WAY OF CONSUMPTION | | PORTION SIZE | | | |
| | | DIVISION | Separately | As part | Small | Medium | Large | |
| | | Almonds | | | | | | |
| | | Hazelnuts | | | | | | |
| | | Walnuts | | | | | | |
| | BEVERAGES | | AMOUNT | | | | | |
| | | DIVISION | Glass/cup | 2x | 3x | 5x | More than 5x | |
| | | Water | | | | | | |
| | | Mineral water | | | | | | |
| | | Coffee | | | | | | |
| | | Coffee with milk | | | | | | |
| | | Tea | | | | | | |
| | | Fresh natural juice | | | | | | |
| | | Soda | | | | | | |
| | | Alcohol | | | | | | |

**COMMENT:**

________________________________________________________________________________

________________________________________________________________________________

| | | | | | | | | | |
|---|---|---|---|---|---|---|---|---|---|
| LUNCH | MEAT | | PREPARATION | | | | PORTION SIZE | | |
| | | DIVISION | Raw | Cooked | Baked | Fried | Small | Medium | Large |
| | | White meat | | | | | | | |
| | | Red meat | | | | | | | |
| | | Fish | | | | | | | |
| | CARBOHYDRATES | | PREPARATION | | | | PORTION SIZE | | |
| | | DIVISION | Raw | Cooked | Baked | Fried | Small | Medium | Large |
| | | Bread | | | | | | | |
| | | Bagels | | | | | | | |
| | | Pasta | | | | | | | |
| | | Potatoes | | | | | | | |
| | | Rice | | | | | | | |
| | VEGETABLES | | PREPARATION | | | | PORTION SIZE | | |
| | | DIVISION | Fresh | Frozen | Cooked | baked | Small | Medium | Large |
| | | Legumes | | | | | | | |
| | | Tuberous/Roots | | | | | | | |
| | | Cabbage | | | | | | | |
| | | Bulbs | | | | | | | |
| | | Pumpkins | | | | | | | |

| | | | | | | | | | |
|---|---|---|---|---|---|---|---|---|---|
| | | ________ | | | | | | | |
| | BEVERAGES | | **AMOUNT** | | | | | | |
| | | **DIVISION** | **Glass/cup** | **2x** | **3x** | **5x** | **More than 5x** | | |
| | | Water | | | | | | | |
| | | Mineral water | | | | | | | |
| | | Coffee | | | | | | | |
| | | Coffee with milk | | | | | | | |
| | | Tea | | | | | | | |
| | | Fresh natural juice | | | | | | | |
| | | Soda | | | | | | | |
| | | Alcohol | | | | | | | |
| | SOUPS | | **PORTION SIZE** | | | | | | |
| | | **DIVISION** | **1x** | **2x** | **3x** | | | | |
| | | Chicken | | | | | | | |
| | | Beef | | | | | | | |
| | | Veggie | | | | | | | |
| | | Cream | | | | | | | |

**COMMENT:**

______________________________________________________________________________

______________________________________________________________________________

| | | | | | | | | | |
|---|---|---|---|---|---|---|---|---|---|
| DINNER | DAIRY PRODUCTS | | **WAY OF CONSUMPTION** | | **PORTION SIZE** | | | | |
| | | **DIVISION** | **Separately** | **As part** | **Small** | **Medium** | **Large** | | |
| | | Cheese | | | | | | | |
| | | Milk | | | | | | | |
| | | Yoghurt | | | | | | | |
| | | __________ | | | | | | | |
| | MEAT | | **PREPARATION** | | | | **PORTION SIZE** | | |
| | | **DIVISION** | **Raw** | **Cooked** | **Baked** | **Fried** | **Small** | **Medium** | **Large** |
| | | White meat | | | | | | | |
| | | Red meat | | | | | | | |
| | | Fish | | | | | | | |
| | | Meat products | | | | | | | |
| | | ________ | | | | | | | |
| | CARBOHYDRATES | | **PREPARATION** | | | | **PORTION SIZE** | | |
| | | **DIVISION** | **Raw** | **Cooked** | **Baked** | **Fried** | **Small** | **Medium** | **Large** |
| | | Bread | | | | | | | |
| | | Bagels | | | | | | | |

| | | | | | | | | | |
|---|---|---|---|---|---|---|---|---|---|
| | | Pasta | | | | | | | |
| | | Potatoes | | | | | | | |
| | | Rice | | | | | | | |
| | FRUIT | | PREPARATION | | PORTION SIZE | | | | |
| | | DIVISION | Fresh | Dried | Small | Medium | Large | | |
| | | Apple/Pear | | | | | | | |
| | | Banana | | | | | | | |
| | | Berries | | | | | | | |
| | | ___________ | | | | | | | |
| | VEGETABLES | | PREPARATION | | | | PORTION SIZE | | |
| | | DIVISION | Fresh | Frozen | Cooked | Baked | Small | Medium | Large |
| | | Legumes | | | | | | | |
| | | Tuberous/Roots | | | | | | | |
| | | Cabbage | | | | | | | |
| | | Bulbs | | | | | | | |
| | | Pumpkins | | | | | | | |
| | | _________ | | | | | | | |
| | CEREALS | | PORTION SIZE | | | | | | |
| | | DIVISION | Small | Medium | Large | | | | |
| | | Oats | | | | | | | |
| | | Corn | | | | | | | |
| | | Sweetened | | | | | | | |
| | | ______________ | | | | | | | |
| | BEVERAGES | | AMOUNT | | | | | | |
| | | DIVISION | Glass/cup | 2x | 3x | 5x | More than 5x | | |
| | | Water | | | | | | | |
| | | Mineral water | | | | | | | |
| | | Coffee | | | | | | | |
| | | Coffee with milk | | | | | | | |
| | | Tea | | | | | | | |
| | | Fresh natural juice | | | | | | | |
| | | Soda | | | | | | | |
| | | Alcohol | | | | | | | |

**COMMENT:**

______________________________________________________________________________

______________________________________________________________________________

| | | | | | | | |
|---|---|---|---|---|---|---|---|
| SNACK | NUTS | | WAY OF CONSUMPTION | | PORTION SIZE | | |
| | | DIVISION | Separately | As part | Small | Medium | Large |
| | | Almonds | | | | | |
| | | Hazelnuts | | | | | |

| | | | | | | | |
|---|---|---|---|---|---|---|---|
| | | Walnuts | | | | | |
| | | ___________ | | | | | |
| | FRUIT | | WAY OF CONSUMPTION | | PORTION SIZE | | |
| | | DIVISION | Fresh | Dried | Small | Medium | Large |
| | | Apple/Pear | | | | | |
| | | Banana | | | | | |
| | | Berries | | | | | |
| | | Citrus | | | | | |
| | | ___________ | | | | | |
| | SWEETS | | PORTION SIZE | | | | |
| | | DIVISION | Small | Medium | Large | | |
| | | Chocolate | | | | | |
| | | Chips | | | | | |
| | | Cookies | | | | | |
| | | Cake | | | | | |
| | | ___________ | | | | | |
| | BEVERAGES | | AMOUNT | | | | |
| | | DIVISION | Glass/cup | 2x | 3x | 5x | More than 5x |
| | | Water | | | | | |
| | | Mineral water | | | | | |
| | | Coffee | | | | | |
| | | Coffee with milk | | | | | |
| | | Tea | | | | | |
| | | Fresh natural juice | | | | | |
| | | Soda | | | | | |
| | | Alcohol | | | | | |

**COMMENT:**

___________________________________________________________________________

___________________________________________________________________________
